# Classification of Wheezing Children in Rural Bangladesh by Intensity of *Ascaris* Infection, Total and Specific IgE Levels, History of Pneumonia, and Other Risk Factors

**DOI:** 10.1155/2019/4236825

**Published:** 2019-12-05

**Authors:** Haruko Takeuchi, Md Alfazal Khan, Khalequz Zaman, Sayaka Takanashi, S. M. Tafsir Hasan, Mohammad Yunus, Tsutomu Iwata

**Affiliations:** ^1^Department of Community and Global Health, Graduate School of Medicine, The University of Tokyo, 7-3-1 Hongo, Bunkyo-ku, Tokyo 113-0033, Japan; ^2^Nutrition and Clinical Services Division, International Centre for Diarrhoeal Disease Research, Bangladesh (icddr,b), 68 Shaheed Tajuddin Ahmed Sarani, Mohakhali, Dhaka 1212, Bangladesh; ^3^Maternal and Child Health Division, icddr,b, 68 Shaheed Tajuddin Ahmed Sarani, Mohakhali, Dhaka 1212, Bangladesh; ^4^Department of Developmental Medical Sciences, Graduate School of Medicine, The University of Tokyo, 7-3-1 Hongo, Bunkyo-ku, Tokyo 113-0033, Japan; ^5^Emeritus Scientist, Maternal and Child Health Division, International Centre for Diarrhoeal Disease Research, Bangladesh (icddr,b), 68 Shaheed Tajuddin Ahmed Sarani, Mohakhali, Dhaka 1212, Bangladesh; ^6^The Graduate School of Humanities and Life Sciences, Tokyo Kasei University, Tokyo, Japan

## Abstract

*Ascaris lumbricoides* is the most common soil-transmitted helminth and infects 447 million people in impoverished areas worldwide. It causes serious morbidity including wheezing and influences various aspects of human immunity, such as type 2 innate lymphoid cells, regulatory T cell function, and acquired immunity. Thus, it is crucial to elucidate its influence on human immunity. We aimed to classify wheezing children based on their *Ascaris* infection intensity and other risk factors using hierarchical cluster analysis to determine the mechanisms of and the degree to which *Ascaris* contributes to childhood wheezing in rural Bangladesh. We analyzed relevant data collected in 2001. The participants included 219 5-year-old wheezing children who were randomly selected from 1705 children living in the Matlab Health and Demographic Surveillance area of the International Centre for Diarrhoeal Disease Research, Bangladesh. Hierarchical cluster analysis was conducted using variables of history of pneumonia, total and specific immunoglobulin E levels, *Ascaris* infection intensity, and parental asthma. Three distinct wheezing groups were identified. Children in Cluster 1 (*n* = 50) had the highest titers of the total, anti-*Ascaris*, anti-*Dermatophagoides pteronyssinus*, and anticockroach IgEs and experienced the fewest episodes of pneumonia. Cluster 2 (*n* = 114), the largest group, experienced few episodes of pneumonia and had the lowest titers of the total, anti-*Ascaris*, anti-Dp, and anticockroach IgEs. Cluster 3 (*n* = 32) consisted of participants with the most episodes of pneumonia and lower titers of the total and specific IgEs. The extremely high prevalence of *Ascaris* infection found in Clusters 1-3 was 78%, 77%, and 72%, respectively. Childhood wheezing in rural Bangladesh could be divided into three groups, with 26% of wheezing attributable to anti-*Ascaris* IgE and 16% to history of pneumonia during early childhood, and 58% might have been due to *Ascaris* infection without elevated anti-*Ascari*s IgE.

## 1. Introduction

*Ascaris lumbricoides* is the most common soil-transmitted helminth (STH), and *Ascaris* infection is one of 13 neglected tropical diseases of great concern. The STH affects approximately 1.5 billion people worldwide, and *Ascaris* infects 447 million people in impoverished areas of Africa, Asia, and Central and South America [1, 2]. The people at risk are preschool children and school-age children [1]. The WHO has implemented a program since 2001 for people at risk in endemic areas in order to eliminate STH infections to reduce intensity of infection and to protect infected individuals from morbidity related to the worms harbored [1]. Although the eradication program of helminthic infections has been on the way, an unacceptably large number of individuals continue to suffer from them despite the program [2]. The morbidity related to the worms harbored includes abdominal pain, general malaise and weakness, intestinal obstruction, and impaired cognitive and physical development. In addition to these symptoms, *Ascaris* causes wheezing; it migrates through the lungs during maturation, where it induces the type 2 inflammatory response, called Löffler's syndrome [3].

A potential explanation for the role of *Ascaris* infection in wheezing might be pulmonary inflammation of type 2 immunity induced by type 2 innate lymphoid cells (ILC2s). Animal worms, such as *Nippostrongylus*, known as the rat hookworm, which have a larval stage in the lungs, have been linked to lung damage, type 2 immune responses, and long-term changes in lung function and structure in nonhuman hosts, which are consistent with allergic airway disease [4]. Migration of *Nippostrongylus* larvae through the lungs causes damage to the epithelium, promoting the release of damage-associated molecular patterns from epithelial cells in the airway [4–6]. The release of interleukin-33 (IL-33) and IL-25 promotes the activation of ILC2s, leading to an increase in the release of the type 2 cytokines, IL-4, IL-5, and IL-13 [4, 6], which have been found to be part of a pathway in both the innate and adaptive responses to lung larval migration in mice [5, 6]. Furthermore, *Ascaris* larval migration causes significant pulmonary damage, including bronchial hyperreactivity (BHR) and type 2 inflammatory lung pathology resembling an extreme form of allergic airway disease in mice [7].

On the other hand, the sharp rise in the worldwide prevalence of bronchial asthma since the 1970s, with children living in industrial and urban areas experiencing higher asthma rates than those in rural area [8–12], has led to the hypothesis that helminthic infections might provide protection against asthma by suppressing the host's immune response. Helminthic infections activate regulatory T cells and induce the production of IL-10, thereby playing a protective role against asthma and allergies. Studies have shown that IL-10 induced in chronic schistosomiasis suppresses atopy in African children [13], and infection with *Schistosoma mansoni* has been associated with a reduced course of asthma [14]. However, we found concurrent decreases in the prevalence of *Ascaris* infection and wheezing from no less than 72% in 2001, to 18% in 2016, and from 16% to 9%, respectively, after implementation of a national deworming program, indicating that the decrease in the prevalence of *Ascaris* infection did not increase wheezing [15].

It appears likely that *Ascaris* infections are associated with increased wheezing. A systematic review and meta-analysis of 22 studies found an association between *Ascaris* infection and wheezing [16]. Another systematic review conducted in Latin America reported an association of a higher risk of asthma or wheezing with an *Ascaris* infestation [17]. However, this relationship remains controversial because the results of multiple epidemiological studies both support and refute the protective effects of helminths on asthma and allergies [13–18].

In 2001, we also reported that anti-*Ascaris* IgE was an increasing risk factor for childhood wheezing in rural areas of Bangladesh [19], and in 2005, we found that anti-*Ascaris* IgE was an increasing risk factor for childhood BHR in the same rural areas [20]. In these studies, *Ascaris* infection itself was not a risk factor for wheezing. The antiparasite role of IgE antibody against helminths is thought to be a normal component of the protective response of the host during infection, and they are not usually associated with allergic symptoms. However, allergic manifestations have been described in some helminth infections such as *Ascariasis* and *Anisakiasis* [21].

*Ascaris* influences on various aspects of human immunity, such as type 2 innate lymphoid cells (ILC2s), Treg function, and acquired immunity; hence, childhood wheezing in rural Bangladesh might be attributable to *Ascaris* infection through a complex interplay between innate, regulatory, and acquired immunity. The mechanism by which *Ascaris* is involved in the development of wheezing and asthma symptoms has caught attention given the serious morbidity caused by this helminth. Therefore, the study's purpose was to classify wheezing children, who participated in the 2001 study, based on the intensity of their *Ascaris* infection, total and specific IgEs including anti-*Ascaris* IgE, parental asthma, and other risk factors to determine the mechanism by which *Ascaris* causes childhood wheezing in this rural area of Bangladesh and the degree to which it contributes to the development of wheezing.

## 2. Methods

The present study reanalyzed the data collected in 2001. The procedures used for the data collection used are described elsewhere [19]. In short, the study population consisted of 1705 5-year-old children randomly selected from Matlab, a riverine rural area located 55 km southeast of Dhaka, the capital of Bangladesh. Children (*n* = 256) who had experienced wheezing during the previous 12 months were identified using a questionnaire adapted from the standardized questionnaire of the International Study of Asthma and Allergies in Childhood (ISAAC) [22]. We retrieved information about the children's history of pneumonia from the routine data-collection system of the International Centre for Diarrhoeal Disease Research, Bangladesh (icddr,b) in the Matlab Health and Demographic Surveillance System (HDSS) area [23]. We also collected blood and stool samples to measure serum total and specific IgEs and to detect helminth infections among the 219 wheezing children whose guardians gave us written informed consent. At that time, the risk factors that were assessed for childhood wheezing included family history of allergies, serum total and anti-*Ascaris* IgE levels, and frequency of pneumonia episodes at 0 years, 1 year, and 2 years of age.

The dataset of the 2001 study included information about wheezing, family history of allergies, socioeconomic status, environmental factors, helminth infections, serum total and antigen-specific IgE levels, and the frequency of pneumonia episodes during the earliest years of childhood. We included the following variables in the present analysis: frequency of pneumonia episodes when the children were 0, 1, and 2 years of age; total, anti-*Ascaris*, anti-*Dermatophagoides pteronyssinus* (Dp), and anticockroach IgE levels; history of parental asthma; and helminth infections.

SPSS 22 (IBM Japan, Tokyo, Japan) was used to perform the cluster analysis with Ward's minimum-variance hierarchical clustering method. The variables were standardized to equalize the standard deviation of the scales. To compare differences among the clusters, analysis of variance (ANOVA) was used for parametric tests of the continuous variables and the Chi-square test was used to analyze the categorical variables. The significance level for all statistical analyses was set at *P* < 0.05.

The Ethics Committee of Tokyo Kasei University approved the study's protocol (Sayama H27-09), and the Ethics Committee of The University of Tokyo approved the protocol (11956). The dataset of the study conducted in 2001 was used in the current study, and the protocol (2000-038) was approved by the Ethical Review Committee of the icddr,b. The 2001 study involved human participants; therefore, it followed the ethical principles of the Declaration of Helsinki. Written informed consent was obtained from the legal guardians of all the participants.

## 3. Results

### 3.1. Characteristics of the Participants

**Figure 1:**
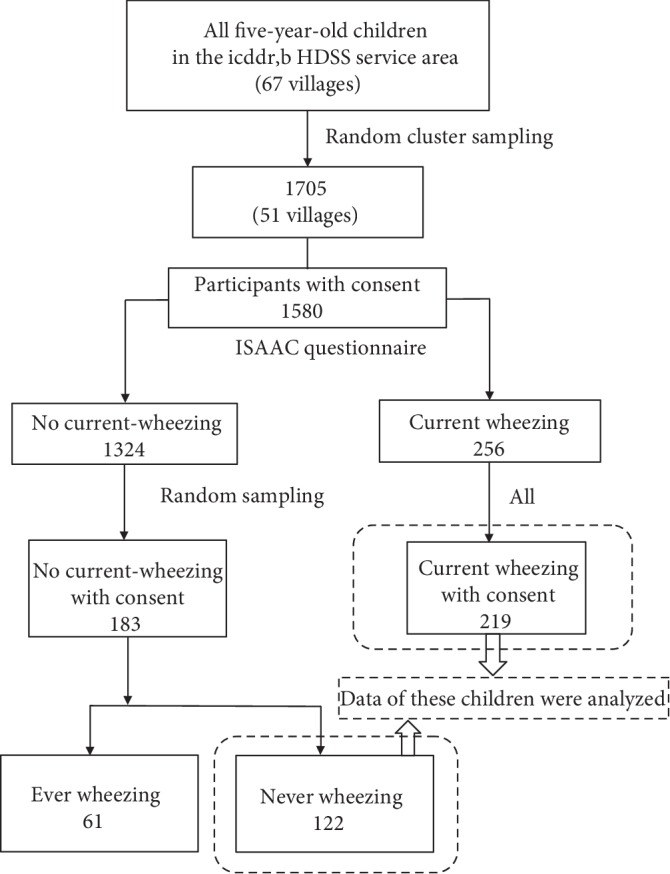
Flowchart of the sampling procedure for the dataset.

The initial study's dataset contained 1705 children who were selected using random-cluster sampling and 1580 of them agreed to participate in the first questionnaire survey. Two hundred fifty-six (16.2%) children were found to have wheezing during the previous 12 months (current wheezing), and 219 participated in the subsequent nested case-control study and submitted blood and stool samples. The information collected from these 219 current wheezing children was used for the cluster analysis. Two hundred fifty-six of the 1324 children with no current wheezing had been randomly selected as the control group and 183 of them agreed to participate in the nested case-control study. The children without current wheezing were divided into two groups. One of the groups consisted of 122 children who had never experienced wheezing (never wheezing) and the other group included 61 children who had experienced wheezing (ever wheezing), but not within the previous 12 months. Data from the 122 children who had never experienced wheezing were used as the comparison group (Figure 1). Tables 1 and 2 show the characteristics of the current- and never-wheezing participants and the three clusters.

### 3.2. Cluster Analysis

**Figure 2:**
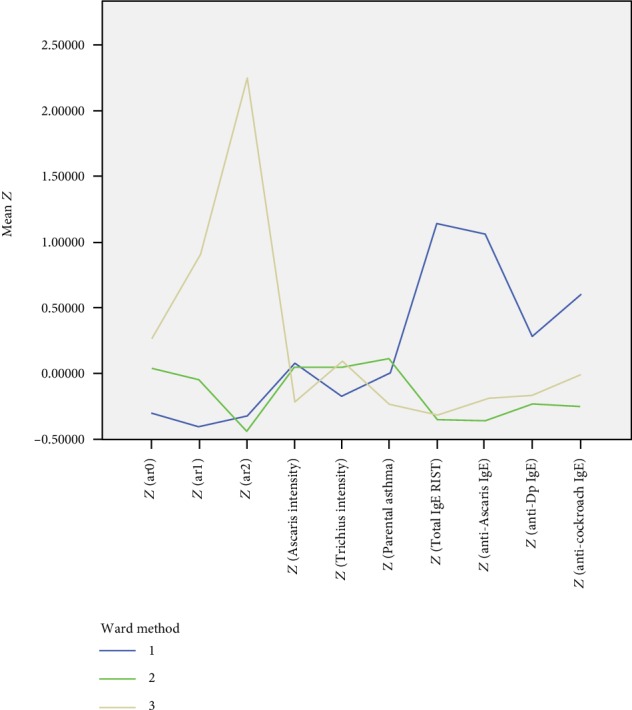
Graph of the three clusters. Three clusters were identified. This polygonal line graph shows the standardized value of the variables regarding the number of the history of pneumonia at 0, 1, and 2 years of age, *Ascaris* infection intensity, *Trichuris* infection intensity, parental asthma, and total and specific IgE levels.

We identified three clusters through the analysis (Figure 2).  Table 1 shows the physical status, family history, and sociodemographic characteristics of the three groups.  Table 2 shows the total and specific IgE levels, prevalence and intensity of *Ascaris* infection, and pneumonia history. The first group consisted of 50 (26%) children who had the highest titers of the total, anti-*Ascaris* IgEs, anti-Dp, and anticockroach IgEs and the lowest frequency of pneumonia episodes. The second group consisted of 114 (58%) children who had a moderate level of pneumonia history and the lowest titers of the total and anti-*Ascaris* IgEs. The third group consisted of 32 (16%) children who had the highest frequency of pneumonia episodes and low IgEs.

#### 3.2.1. Cluster 1

Twenty-six percent of the participants (*n* = 50) were grouped into Cluster 1. This cluster was characterized by significantly high serum levels of the total and anti-*Ascaris* IgE levels, and high anti-Dp and anticockroach IgE levels, and a significantly lower number of children with a history of pneumonia when they were 0, 1, and 2 years of age. A significantly lower number of the mothers in Cluster 1 were educated at the primary level, and their household income (monthly) was lower than that of the households in the other groups, although the difference was not significant. We found no significant differences from other groups, with respect to sex, physical measurements, parental history of asthma, or type of cooking fuel. Cluster 1 had the highest prevalence and intensity of *Ascaris* infection (78%) although the differences between the three groups were not significant.

#### 3.2.2. Cluster 2

Cluster 2 was the largest group (*n* = 114; 58%) of participants. It consisted of children with a moderate frequency of pneumonia episodes and the lowest serum levels of the total, anti-*Ascaris*, anti-Dp, and anticockroach IgE, which were comparable to the levels of the never-wheezing children. Their mothers had significantly more education, a relatively high household income (monthly), and a higher prevalence of asthma than the mothers did in Cluster 3, although the differences were not significant. The prevalence (77%) and intensity (37.7%) of *Ascaris* infection were as high as Cluster 1.

#### 3.2.3. Cluster 3

Cluster 3, which was the smallest cluster (*n* = 32; 16%), was characterized by their frequency of pneumonia episodes when they were 0, 1, and 2 years old. They experienced a significantly higher frequency of pneumonia episodes than the children in the other groups did. They had more *Trichuris* infections, and their mothers had a lower rate of asthma than the mothers did in the other two groups, although the differences were not significant. The prevalence (71.9%) and intensity (21.9%) of *Ascaris* infection were lower in Cluster 3 than in the other two groups and were almost comparable to the never-wheezing group (71.6% and 29.4%, respectively).

## 4. Discussion

The major finding of this analysis was that three distinct clusters of wheezing children in rural Bangladesh were identified, with children having a high titer of anti-*Ascaris* IgE comprising Cluster 1. Participants in this group (*n* = 50) had higher titers of the total IgE, anti-*Ascaris* IgE, anti-Dp IgE, and anticockroach IgE levels and experienced fewer episodes of pneumonia. Children in Cluster 2 (*n* = 114) had a low frequency of pneumonia episodes and lower titers of the total, anti-*Ascaris*, anti-Dp, and anticockroach IgE. Cluster 3 (*n* = 32) consisted of children with a higher frequency of pneumonia episodes and lower titers of the total and specific IgEs. The prevalence of *Ascaris* infection was high in all clusters (78%, 77.2%, and 72%), and it was higher in Clusters 1 and 2 than in Cluster 3, although the differences were not significant.

The children in Cluster 1 had higher titers of the total and anti-*Ascaris* IgE and slightly elevated anti-Dp and anticockroach IgE levels. We reported that in 2005, elevated serum anti-*Ascaris* IgE was associated with BHR in children in rural Bangladesh [20]. This finding was supported by a subsequent study, which was conducted in the same region of Bangladesh in 2008, when the infection prevalence was 17.4%. That study reported that anti-*Ascaris* IgE was associated with an increased risk of ever having asthma among 5-year-old children [24]. Studies conducted in Latin America have also reported similar results [25–27]. The fact that children in Cluster 1 had higher titers of anti-Dp and anticockroach IgE may be explained by a predisposition to atopy among the children in this group. In other words, the children in Cluster 1 are likely to produce high titers of anti-*Ascaris* IgE when they were infected with *Ascaris* because they were atopic. This group may resemble to multisensitized atopic wheezing cluster in other studies [28]. However, neither the family history of asthma nor allergies were obvious in Cluster 1.

Another explanation for the elevated levels of anti-*Ascaris* and anti-Dp IgE is the cross-reactivity between the *Ascaris* and the house-dust mite antigens. The *Ascaris* antigen's cross-reactivity with that of the house-dust mite through tropomyosin might stimulate the production of elevated anti-Dp and anticockroach IgE [29]. Therefore, if *Dermatophagoides* antigen is abundant in the environment and is inhaled, it might join with anti-*Ascaris* IgE on the mast cell surface of the airway and result in wheezing [30, 31]. It is understandable that the group with a high level of anti-*Ascaris* IgE comprises one cluster, as anti-*Ascaris* IgE was an independent risk factor for wheezing [19]. In the study in 2001, whose participants are the target population of the present study, the odds ratios of anti-*Ascaris* IgE levels for current wheezing increased and *P* values decreased as the children expressed severer symptoms. This association was not found in total, anti-DP, or anticockroach IgEs.

The children in Cluster 2 experienced relatively few episodes of pneumonia and had the lowest titers of the total and anti-*Ascaris* IgE and the lowest anti-Dp IgE level. Before the analysis, we expected to find an association of family history of asthma and allergies with a high titer of anti-Dp IgE in this group or with *Ascaris* infection intensity as measured by *Ascaris* egg count in the stool. Therefore, we analyzed *Ascaris* infection intensity and family history asthma and allergies by the demographic and health-related characteristics of the three groups: sex; history of diarrhea; physical status; number of family members; number of older or younger children; number of rooms in the house; duration of exclusive breast feeding; coverage for the diphtheria, pertussis, and tetanus vaccine and the measles and bacillus Calmette-Guerin vaccines; eczema; allergic rhinitis; household smoking; water supply; and parental education. However, no specific characteristics of the children were found to be significant in the analysis, except for a higher level of maternal education. Then, what factor contributed to wheezing in Cluster 2? This group and Cluster 1 had a higher prevalence of *Ascaris* infection than did Cluster 3, although the difference was not significant. The main difference between Clusters 1 and 2 was the serum levels of IgE, indicating that children in Cluster 1 had the capacity to produce high titers of IgE than Cluster 2. In 2015, we conducted an epidemiological study, which found concurrent decreases in the prevalence of wheezing and *Ascaris* infection among 5-year-old children in rural Bangladesh [15]. The study also showed that wheezing children had a significantly higher rate of *Ascaris* infection compared to never-wheezing children, although *Ascaris* infection was not a risk factor for wheezing. However, it was evident that wheezing and the prevalence of *Ascaris* infection decreased simultaneously.

In animal models, worms have been linked to type 2 immune responses through ILC2s in the lungs, including airway hyperresponsiveness which resembles an extreme form of allergic airway disease [4–7]. Although the function of human ILC2s in *Ascaris* infection should be investigated epidemiologically and experimentally in future studies, it has been reported that *Ascaris* induces an inflammatory response in the lungs independent of its effect on IgE production, which may explain some of the contradictory findings of studies examining the association between geohelminth infection, atopy, and asthma [18]. As anti-*Ascaris* IgE increases only in individuals with current or past *Ascaris* infections, the notion that childhood wheezing in rural Bangladesh might be attributable to *Ascaris* infection is reasonable. These findings indicate that the high prevalence of *Ascaris* infection in Clusters 1 (78%) and 2 (77.2%) might be a contributing factor to the wheezing of the children in these groups.

Cluster 3 (*n* = 32) consisted of children with a higher frequency of pneumonia episodes and lower titers of the total and specific IgEs. In the present study, information about pneumonia episodes was obtained from the record-keeping system of the HDSS of the icddr,b. In 2001, pneumonia was recorded by community health research workers every two weeks. This surveillance was based on the mother's report of the child's increased respiratory rates with or without chest indrawing, following the World Health Organization's guidelines [32]. *Haemophilus influenzae*, *Streptococcus pneumoniae*, *Branhamella catarrhalis*, and Gram-negative bacilli were the predominant causative bacteria of pneumonia in 157 patients who were admitted to a pediatric hospital for treatment of pneumonia, as reported in a 1998 study conducted in Dhaka, Bangladesh [33]. Respiratory syncytial virus, which is known to cause recurrent wheezing and BHR in later life [34, 35], was the most common virus detected in children less than 2 years old who were hospitalized due to severe lower respiratory tract infections. Rhinovirus, a causative agent of the common cold, is also related to exacerbations of asthma attacks in 80% of children and might have been present in 50% of adults [36, 37]. Although we did not examine the etiologic agents of pneumonia in our study, we speculate that the majority of episodes might have been due to these pathogens.

Acute lower respiratory infections (ALRI) have been major causes of morbidity and mortality in Bangladesh; however, improvements in the management of childhood illnesses have successfully decreased deaths caused by ALRI among young children [38]. Thus, it is understandable that these children had a higher risk of developing asthma in subsequent years. The symptoms of children in Cluster 3 were compatible with these observations, indicating the need for attention to wheezing post-ALRI in order to stem the increase in asthma in rural Bangladeshi children. This group might be comparable to the nonatopic postviral bronchial hyperresponsiveness group of Tucson Study [39].

We found 3 clusters as predictive index for asthma in infants and preschoolers in rural Bangladesh. Anti-*Ascaris* IgE was an independent risk factor for wheezing against the fact that anti-DP IgE was not a risk factor for wheezing. However, children with high anti-*Ascaris* IgE and anti-Dp IgE seem to comprise 1 group, which indicates that the children with high anti-*Ascaris* IgE might emerge as children who have high anti-Dp IgE with the development of the society in the future. Since the children in this group might develop persistent wheezing in the future through early sensitization by any antigen, early sensitization with *Ascaris* antigen by *Ascaris* infection should absolutely be prevented. Therefore, we think children with high anti-*Ascaris* IgE might need to be followed up carefully, before the development of the future atopic type to curb the increase of persistent asthma.

## 5. Conclusion

In conclusion, data on childhood wheezing from a study conducted in 2001 was classified into three distinct categories; 26% of the wheezing was attributable to anti-*Ascaris* IgE, 16% to the history of pneumonia during early childhood, and the remaining 58% might have been due to *Ascaris* infection. Although we could not find any specific characteristics in Cluster 2, we speculate that the high prevalence of *Ascaris* infection might have been a contributing factor to wheezing. Childhood wheezing caused by *Ascaris* infection might be induced through the complex interplay between innate, acquired, and regulatory immunity, although the underlying mechanism for such wheezing remains unclear. As *Ascaris* infection remains a major public health problem in this rural area of Bangladesh, despite its dramatic decrease in prevalence, the role of ILC2s, anti-*Ascaris* IgE, and Tregs in *Ascaris* infection on childhood wheezing merits further investigation.

## Figures and Tables

**Table 1:** Comparisons of physical status, family history, and sociodemographic characteristics of the three groups.

	Total	Cluster 1	Cluster 2	Cluster 3		Never-wheezing
*n*	219	50	114	32	*P*=	122
Sex, female (%)	108 (49)	22 (44)	63 (55)	12 (38)	0.138	66 (49)
Physical measurements (*n*)	194	49	113	32		122
Height (cm)	103.0	102.4	103.6	102.3	0.213	103.7
Weight (kg)	14.7	14.5	14.8	14.5	0.492	14.8
DPT3 vaccine coverage (%)	185 (94.4)	45 (90.0)	108 (94.7)	32 (100)	0.154	
Measles vaccine coverage (%)	188 (95.9)	47 (94.0)	109 (95.6)	32 (100)	0.395	
Family history						
Mother's asthma (%)	42 (19)	11 (22.0)	26 (22.8)	2 (6.3)	0.106	12 (9.8)
Father's asthma (%)	25 (12)	4 (8.2)	16 (14.0)	4 (12.5)	0.578	2 (1.7)
Dry leaves as fuel	183 (86)	42 (89)	93 (83)	28 (88)	0.548	91 (75)
Mother's education (none) (%)	93 (43)	29 (58)	41 (36)	17 (53)	0.018	48 (39)
Monthly income (BTk)	3943	2896	4164	3712	0.054	4755

DPT3: diphtheria, pertussis, tetanus vaccine; BTk: Bangladesh Taka.

**Table 2:** Comparisons of serum IgE levels, helminth infections, and pneumonia history among the three groups.

	Total	Cluster 1	Cluster 2	Cluster 3		Never-wheezing
*n* (%)	196 (100)	50 (25.5)	114 (58.2)	32 (16.3)	*P*=	122
Total IgE (IU/ml)		13598	3705	3959	<0.001	3686
Specific IgE (U_A_/ml)						
Anti-*Ascaris* IgE	30.8	62.5	20.3	24.8	<0.001	14.9
Anti-Dp IgE	4.1	7.8	1.8	2.7	<0.001	1.8
Anticockroach IgE	4.2	8.1	2.3	4.0	<0.001	2.8
Helminth infection	199					
*Ascaris* egg (+) (%)	152 (76.4)	39 (78.0)	88 (77.2)	23 (71.9)	0.789	78 (71.6)
(+++) (%)	71 (35.7)	21 (42.0)	43 (37.7)	7 (21.9)	0.158	32 (29.4)
*Trichuris* (+) (%)	100 (50.3)	22 (44.0)	56 (49.1)	20 (62.5)	0.252	66 (60.6)
Pneumonia history (+) *n* (%)						
At 0 years	56 (25.6)	6 (12.0)	31 (27.2)	12 (37.5)	0.024	16 (13.1)
1 year	44 (20.1)	2 (4.0)	21 (18.4)	18 (56.3)	<0.001	4 (3.3)
2 years	38 (16.4)	2 (4.0)	0 (0.0)	32 (100)	<0.001	2 (2.0)

IgE: immunoglobulin E; Dp: *Dermatophagoides pteronyssinus*.

## Data Availability

The numeric-type data used to support the findings of the current study are available from the corresponding author upon reasonable request.
